# Converting Organic Municipal Solid Waste Into Volatile Fatty Acids and Biogas: Experimental Pilot and Batch Studies With Statistical Analysis

**DOI:** 10.2196/50458

**Published:** 2025-02-04

**Authors:** Hojjat Borhany

**Affiliations:** 1 Faculty of Environmental Science, Department of Environmental Science, Informatic, and Statistics, University of Ca' Foscari Venice, Mestre (VE), Italy

**Keywords:** multistep fermentation, specific methane production, anaerobic digestion, kinetics study, biochar, first-order, modified Gompertz, mass balance, waste management, environment sustainability

## Abstract

**Background:**

Italy can augment its profit from biorefinery products by altering the operation of digesters or different designs to obtain more precious bioproducts like volatile fatty acids (VFAs) than biogas from organic municipal solid waste. In this context, recognizing the process stability and outputs through operational interventions and its technical and economic feasibility is a critical issue. Hence, this study involves an anaerobic digester in Treviso in northern Italy.

**Objective:**

This research compares a novel line, consisting of pretreatment, acidogenic fermentation, and anaerobic digestion, with single-step anaerobic digestion regarding financial profit and surplus energy. Therefore, a mass flow model was created and refined based on the outputs from the experimental and numerical studies. These studies examine the influence of hydraulic retention time (HRT), pretreatment, biochar addition, and fine-tuned feedstock/inoculum (FS/IN) ratio on bioproducts and operational parameters.

**Methods:**

VFA concentration, VFA weight ratio distribution, and biogas yield were quantified by gas chromatography. A *t* test was then conducted to analyze the significance of dissimilar HRTs in changing the VFA content. Further, a feasible biochar dosage was identified for an assumed FS/IN ratio with an adequately long HRT using the first-order rate model. Accordingly, the parameters for a mass flow model were adopted for 70,000 population equivalents to determine the payback period and surplus energy for two scenarios. We also explored the effectiveness of amendments in improving the process kinetics.

**Results:**

Both HRTs were identical concerning the ratio of VFA/soluble chemical oxygen demand (0.88 kg/kg) and VFA weight ratio distribution: mainly, acetic acid (40%), butyric acid (24%), and caproic acid (17%). However, a significantly higher mean VFA content was confirmed for an HRT of 4.5 days than the quantity for an HRT of 3 days (30.77, SD 2.82 vs 27.66, SD 2.45 g–soluble chemical oxygen demand/L), using a *t* test (*t*_8_=−2.68; *P*=.03; CI=95%). In this research, 83% of the fermented volatile solids were converted into biogas to obtain a specific methane (CH_4_) production of 0.133 CH_4_-Nm^3^/kg–volatile solids. While biochar addition improved only the maximum methane content by 20% (86% volumetric basis [v/v]), the FS/IN ratio of 0.3 volatile solid basis with thermal plus fermentative pretreatment improved the hydrolysis rate substantially (0.57 vs 0.07, 1/d). Furthermore, the biochar dosage of 0.12 g-biochar/g–volatile solids with an HRT of 20 days was identified as a feasible solution. Principally, the payback period for our novel line would be almost 2 years with surplus energy of 2251 megajoules [MJ] per day compared to 45 years and 21,567 MJ per day for single-step anaerobic digestion.

**Conclusions:**

This research elaborates on the advantage of the refined novel line over the single-step anaerobic digestion and confirms its financial and technical feasibility. Further, changing the HRT and other amendments significantly raised the VFA concentration and the process kinetics and stability.

## Introduction

The European Union annually generated about 110 million tons of organic waste in 2006, which excluded slurry and manure. This waste mainly came from the food industry (33%), agriculture and hunting (30%), and households (20%) [1]. Current Italian legislation forbids landfilling organic waste and requires treating it through biological and thermal processes like anaerobic digestion, composting, and incineration with high disposal costs for secondary waste flux (€75‐€125 per ton; a currency exchange rate of €1=US $1.05 is applicable) [2]. Under the pressure of exhaustible natural exploitation and increasing organic waste, the European Commission approved the circular economy action plan to promote sustainable recovery methods to reduce the secondary waste flux. The techniques recommended in the circular economy context assume a “take-use-reuse” viewpoint. Such an approach wants to close the circuit of cycles, extend product life, and treat the wastes as precious recyclable materials [3,4]. In this respect, the European Union states have deployed biological processes such as anaerobic digestion to gain either platform chemicals like volatile fatty acids (VFAs) or biogas from organic wastes produced in urban areas [5-9]. These products are extremely valuable in the era of environmental disasters, which have several consequences (eg, climate change), since they are renewable, sustainable, carbon-neutral, and compatible with current fossil-based fuel infrastructures [10].

Recent studies have aimed at finding a sequential reclaiming route to obtain various bioproducts such as VFAs and biohydrogen with a higher added-value market than bio-methane at distinct steps to either redesign the existing plants or integrate them into biorefinery platforms [11,12]. Various biological processes can convert different feedstock (eg, edible sugary crops, oil-bearing crops, livestock, waste sludge [WS], and food waste) into a range of biofuels, including bioethanol, biodiesel, bio-methane, and biohydrogen [10,13,14]. Biofuel production from edible crops is quite controversial in terms of food supply, ethical quandary, and insecure supply chain. However, food waste, WS, and livestock are omnipresent in urban and rural areas without widespread deployment in a biorefinery scheme. Accordingly, this research aims to convert organic municipal solid waste (OMSW), mainly from food waste, into VFAs and biogas.

This study examines the biological recovery route for OMSW for potential beneficial bioproducts and technical feasibility. This effort includes three steps: pretreatment, mesophilic acidogenic fermentation, and anaerobic digestion. Specifically, we endeavor to conceive how to make the process more profitable and practicable through operational amendments that change the share of methanogenesis and acidogenic routes in the final products (VFAs and biogas) [9] and lower the costs of the process in terms of energy and water consumption. Hence, determining a reasonably priced process with a desirable VFA-rich stream from acidogenic fermentation and a high methane (CH_4_) yield from methanogenesis [15] could ultimately encourage full-scale commercialization. VFAs typically serve as platform chemicals for many processes (eg, biopolymer synthesis of polyhydroxyalkanoates [PHAs] [16-19]), which could be later recovered through biological processes to close the material life cycle.

The major bottleneck in anaerobic digestion of biowaste is at the hydrolysis step. Such a problem could be relieved by various methods such as pretreatment, an optimized feedstock/inoculum (FS/IN) ratio, and carbonaceous material addition, including biochar [20-22]. The latter method was recently realized to have numerous benefits to the process, such as improving the process stability, acceleration of the process rate, buffering potency and alkalinity, inhibitors adsorption, enriched microbial functionality, and electron transfer mechanism. As a result, it could improve CH_4_ generation by fostering hydrolysis, acetogenesis, and methanogenesis [23]. The residual solids out of the multistep line of pretreatment followed by acidogenic fermentation plus anaerobic digestion can be used in a pyrolysis line for biochar and biofuel production to further lower the secondary waste flux [24]. This strategy provides several benefits, such as combating climate change and global soil degradation and addressing the rising energy demand.

This study compares the multistep route of pretreatment, acidogenic fermentation, and anaerobic digestion with the existing method of single-step anaerobic digestion for valorizing OMSW in the Treviso wastewater treatment plant (WWTP) in terms of financial profit and technical feasibility. In this context, the present research has the ultimate goals of facilitating the entrance of the process into the market and further closure of the cycle of organic material. Accordingly, it assesses several suggestions, such as hydraulic retention time (HRT) variation, pretreatment, biochar addition, and adjusted FS/IN ratio to enhance the bioproducts and decrease the involved costs. To this end, their effects on the process were quantified through experimental tests, confirming their significance through statistical analysis. Later, the payback period, amount of surplus energy, and volatile solids (VS) destruction for the mentioned scenarios were determined using a mass balance model refined according to the laboratory studies. The boundary condition parameters for energy conversion and costs were assumed according to previous studies and experts’ knowledge, respectively. To the best of the author’s knowledge, this paper is novel in presenting a robust framework to assess a groundbreaking proposition for the valorization of OMSW financially and technically. Overall, we concluded that our line is viable technically and overtakes the conventional methods financially.

## Methods

### Biorefinery Process Scheme and Experimental Studies

**Figure 1.**
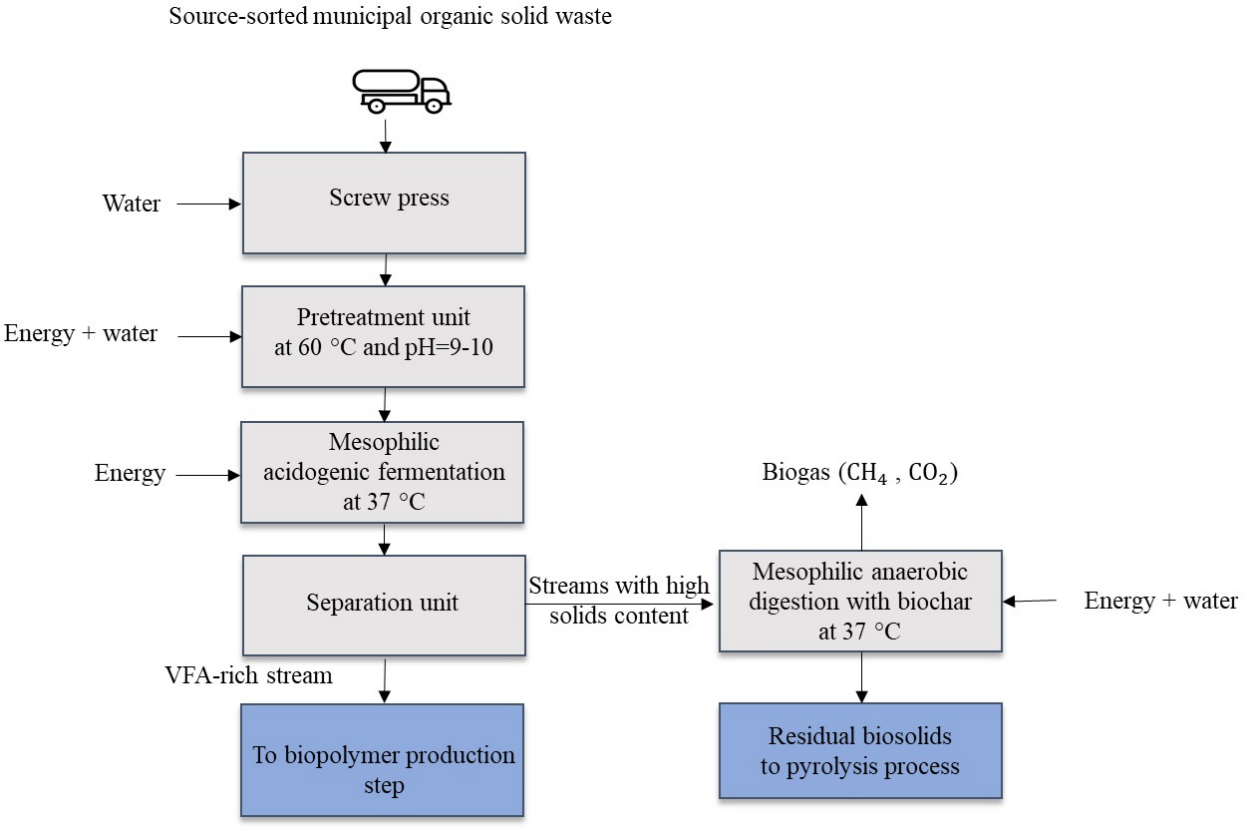
Schematic of the multistep process of pretreatment, acidogenic fermentation, and anaerobic digestion for VFAs and biogas production from the organic municipal solid waste. CH_4_: methane; CO_2_: carbon dioxide; VFA: volatile fatty acid.

Figure 1 presents the hypothesized biorefinery process line in this research. It comprises screw-pressing, a pretreatment unit, mesophilic acidogenic fermentation, solid-liquid separator, and mesophilic anaerobic digestion. The two sectors of biopolymer production and pyrolysis were exhibited differently since no mass and energy flow was considered for them, and only the possible end goals for the secondary stream were shown.

After and before the pretreatment, the feedstock for different parameters was characterized from time to time. These parameters include the total solids (TS), VS, chemical oxygen demand (COD), soluble COD (SCOD), total Kjeldahl nitrogen, total phosphorous (P), ammonium (N-NH_4_^+^), phosphate (P-PO_4_^3-^), and VFA.

The feedstock that arrived at the WWTP had already been mixed with the acidogenic fermentative inoculum, which initiated solubilizing and converting the organic solid matters into SCOD and VFAs in the transporter. Then, in the pretreatment unit, a sodium hydroxide (NaOH) solution (40% kg/kg) was added to bring the pH to 9‐10 and heated to 60 ℃ for 24 hours. Subsequently, the biomixture was fed manually into a 5 L (operational volume of 4.5 L) continuously stirred pilot acidogenic fermenter operated at the given conditions (Table 1). Its high alkalinity maintained the pH during the acidogenic fermentation in the optimal range. Further, the mixture was blended mechanically, and the whole system was kept in the oven to hold the temperature constant at 37 ℃. The output was sampled frequently during the week, and the samples were centrifuged to obtain the supernatant to measure pH, SCOD, VFA, N-NH_4_^+^, and P-PO_4_^3-^. A tiny fraction of the residual solid part was used to characterize solids like COD, P, and total Kjeldahl nitrogen, and the rest was kept in the freezer to apply the bio-methane potential (BMP) test.

**Table 1.** The operational parameters of the mesophilic acidogenic fermenter.

Hydraulic retention time (days)	Organic loading rate (kg–volatile solids/m^3^.d)	Temperature (℃)	pH^a^, mean (SD)
4.5	6.89	37	6.56 (0.25)
3	10.33	37	6.7 (0.45)

a 13 measurements for pH.

The VS and TS characterization were performed in 105 ℃ and 550 ℃ ovens for 24 hours, respectively. Except for VFAs, all the remaining analyses (including COD measurements) followed the standard methods for examining water and wastewater [25]. The methods described in the A and D sections of No. 5220 for COD quantification were used. These methods are named “Closed Reflux, Titrimetric Method” and “Closed Reflux, Colorimetric Method” for the solid and liquid phases, respectively [25]. For the liquid, the samples were filtered after being centrifuged at 4500 rounds per minute (rpm) for 5 minutes, and before the analysis, the supernatant was filtered with a 0.45 μm cellulose filter (Whatman). For the solid, acidic digestion was performed at 220 ℃ with a high pressure of 2 atmospheres to destroy the 0.2 g of solid matrix for 2 hours. Afterward, the COD was measured in the solution using titration by ferrous ammonium sulfate as described in the standard methods. Our limit of detection was 50‐500 mg-COD/L for the calorimetric method and 40‐400 mg-COD/L for the titrimetric method. In this research, dilution was done for high-concentration values that are beyond the considered limit of detection.

In the BMP test, the effect of biochar addition was observed for 3 diverse dosages (0, 0.12, and 0.24 g-biochar/g-VS) on the bio-methane volume, content, and production kinetics in the mesophilic condition using four sets of the BMP test. The tests were conducted with a total number of 8 bottles of 250 mL (working volume of 215 mL). The anaerobic condition was ensured in bottles by sealing them after filling without any flushing with nitrogen molecules (N_2_) or carbon dioxide (CO_2_) since we had known that oxygen transfer at the surface of the waste stream was impossible as it contained a high TS and SCOD. This type of procedure was adopted in our laboratory and has been conducted for years. The biochar was synthesized by a local supplier, and its main physical and chemical features are reported in Table S1 in Multimedia Appendix 1. It was ground into microparticles and kept under a dried condition at room temperature before being added to the bottles. Further, the inoculum for the BMP test was collected from the 2300 m^3^ completely stirred anaerobic digester treating thickened WS and squeezed OMSW mixture under the mesophilic condition at an organic loading rate (OLR) of 1.8‐2.0 kg-VS/m^3^.d in the treatment plant. The inoculum was added to the feedstock (residual solid from acidogenic fermentation) based on the weight ratio of 0.3 FS g-VS/IN g-VS. The TS and VS contents in the bottles (ie, inoculum and feedstock) were 133 g/kg and 17.6 g/kg, respectively.

The experiments were conducted for each condition, namely, only inoculum and either with or without biochar, in 2 bottles. The test was terminated after 25 days when the cumulative biogas production reached almost 89% of the final projected value. The biogas content was characterized by gas chromatography (for days 1, 4, 6, 10, 14, 16, 18, 21, and 25). Additionally, the values for the remaining days were filled through imputation using the *k*-nearest neighbors algorithm (number of neighbors=4 and weights=distance) [26]. The imputation code is provided in the repository [27]. Then, the biogas and bio-methane volumes were subtracted from the only inoculum to correct for the endogenous methane production, and both values were averaged for 2 bottles. Gas chromatography was performed using Agilent Technology (TM 6890N) with an HP-PLOT MoleSieve column (30 m length, 0.53 mm ID × 25 mm film thickness) and a thermal conductivity detector with argon as a carrier (79 mL/min). The hydrogen molecule (H_2_), CH_4_, oxygen molecule (O_2_), and N_2_ were analyzed using a thermal conductivity detector at 250 ℃. The inlet temperature was 120 ℃, with constant pressure in the injection port (ie, 70 kilopascal [kPa]). Samples were taken using a gas-type syringe (200 µL). Once the entire sample was vaporized, peak separation occurred within the column at a constant temperature of 40 ℃ for 8 minutes. We did not plan to monitor pH and other parameters like alkalinity, VFA, ammonia, and phosphate because the pH drop risk was negligible, and the biochar addition could provide a buffer capacity and adsorption of inhibitory compounds in the solution [28]. Moreover, a considerable part of the readily biodegradable COD of the feedstock was already converted to VFAs in the previous step. As a result, the process was easily controlled even in the transient condition when the risk of methanogenic inhibition was high [29].

### Statistical Analysis and Performance Indicators

The performance indicators, including COD solubilization, VFA yield, ammonia and phosphate release, and VFA/SCOD ratio were determined. These indicators characterize the mesophilic acidogenic fermentation on the days when the data were available, and the process reached the pseudo-steady state condition. The indicators were calculated, and the data were plotted using a Microsoft Excel spreadsheet (Version 2412). In addition, the VFA weight ratio distribution was determined from the total VFA weight on the same day. The process stability was evaluated based on variations in daily VFA concentrations. The formula for the performance parameters is reported in Multimedia Appendix 1. The exploratory data analysis and 2-tailed *t* test on VFA data were performed for the VFA concentration, yield, and VFA/SCOD ratio for 2 HRTs by the open source program R (version 3.5.0; The R Foundation for Statistical Computing). We assumed that the 2 datasets were paired and had a normal distribution. The code is provided in the repository [27]. The values for the 2 HRTs to increase the VFA concentration in the outlet were selected based on our experience and process knowledge. According to this information, exceeding the HRT value by more than 3‐5 days can bring the process into an anaerobic digestion step. As a result, the VFAs with high added-value markets are converted to biogas. Hence, the 2 HRTs of 3 days and 4.5 days were tried in the pilot test, knowing that the VFA concentration would either increase or decrease linearly in this local region of operation.

For the BMP tests, two kinetic models were calibrated, namely, the first-order rate and modified Gompertz, to the biogases’ cumulative yield. Additionally, the specific methane production (SMP) and specific biogas production (SGP) plus maximum volumetric methane content (v/v %) were determined. Comparing these results could reveal how the biochar addition, FS/IN ratio of 0.3, and pretreatment improved the process in terms of the rate and fostered methanogenesis. Such improvements are manifested through a higher hydrolysis rate, a shorter lag phase, and a higher maximum volumetric methane content. Besides, the biogas yield was determined as g-biogas/g-VS.

### Technical and Economic Assessment

This research sets up a mass flow analysis with parameters adopted for a municipality with 70,000 population equivalents (PEs) for the two scenarios: (1) a line with pretreatment and mesophilic acidogenic fermentation followed by mesophilic anaerobic digestion and (2) a single-stage mesophilic anaerobic digestion as currently deployed at the Treviso WWTP. This study focuses on water and energy preservation and increased profits from VFA production in our conversion line through several refinements. They were tied with the HRT identified in the previous step, integration of our process knowledge of using the fine-tuned FS/IN ratio, and biochar addition in anaerobic digestion. Detailed information and calculations regarding the mass flow analysis are available in the supplementary documents in the Excel spreadsheet named “Mass Balance” [27]. The following paragraph provides the full description of the two scenarios.

The two scenarios shared the first part of the model where the separated OMSW by a door-to-door collection system that was screw-pressed and diluted with water to reach the TS of 280 g/kg. Then, in the first scenario, adding a sodium hydroxide solution (40% kg/kg) elevated the feedstock pH to 9‐10. Afterward, the solution was heated at 60 ℃ for 24 hours in the pretreatment unit. Next, it was diluted and heated further before feeding into the mesophilic acidogenic fermenter based on the desirable HRT. The last part of the first scenario was the optimized anaerobic digestion of residual fermented solids. Specifically, the stability endowment by adding biochar to the anaerobic digestion could ultimately smooth running the process in a high OLR (low water dilution). Furthermore, an FS/IN ratio of 0.3 was applied to increase the kinetics rate with the benefit of a decrease in digester volume, energy consumption, and capital cost. This finding is of significant importance in plants and zones with limited area, water, and energy.

In the second scenario, the screw-pressed feedstock was diluted and immediately fed into a mesophilic anaerobic digester for only biogas production.

It was assumed that the reactors transfer heat from the walls with the atmosphere and earth. Further, the biogas would be consumed in the combined heat and power units for electricity production with an overall efficiency of 0.4. In this research, the mass of VFAs and the net amount of energy production were accounted for as the source of income. Meanwhile, the corresponding costs were the operational expenditure, the mass of the water process, and the final residual solids to dispose of. Reference parameters for the energy analysis and boundary conditions are given in Table 2. The price of electricity was assumed to be €130 per megawatt-hour (MWh). These two scenarios were compared to identify the most favorable one regarding surplus thermal energy and electricity or the shorter payback period.

**Table 2.** Reference parameters and boundary conditions for energy flow analysis.

Parameter	Heat transfer coefficient (W/(m^2^.℃))	Temperature (℃)	Low heat value (MJ^a^/Nm^3^)	Energy conversion efficiency
Biogas	—^b^	—	23.012	—
Thermal energy yield	—	—	—	0.5
Electrical energy yield	—	—	—	0.4
Operative temperature	—	37	—	—
Water temperature	—	15	—	—
Air temperature	—	20	—	—
Ground temperature	—	25	—	—
Outer concrete reactor wall	0.7	—	—	—
Inner concrete reactor wall	1.2	—	—	—
Floor	2.85	—	—	—

a MJ: megajoules.

b Not applicable.

### Ethical Considerations

This research was not conducted on human or animal subjects and does not involve the collection of any new data. Therefore, it was unnecessary to obtain ethics approval.

## Results

### Biorefinery Process Scheme and Experimental Studies: Composition and Characteristics of the Pretreated Feedstock

The pretreated feedstock’s main physical and chemical characteristics were quite stable throughout the experiment (Table 3). The feedstock had an average TS content of 45 (SD 3.15) g/kg and VS content of 32 (SD 3.28) g/kg. These values suggest that the biodegradable solids constituted 72% of the TS, which could support the fermentation process. The chemical composition of the solid part was 12.9 g-N/kg-TS, 4 g-P/kg-TS, and 565 g-COD/kg-TS, which was in the range of the values reported for the typical OMSW in Italy [30]. The chemical composition of the liquid was 325 mg N-NH_4_^+^/L, 14 mg P-PO_4_^3-^/L, and 25.8 g-SCOD/L. Further, the feedstock COD:N:P ratio was determined as 100:2.2:0.7, meaning that nutrients such as phosphor and nitrogen should not be the limiting substrates in acidogenic fermentation [31]. In this regard, the slight level of VFA concentration at the level of 3.5 g-SCOD/L was due to acidogenic fermentation, which had been happening during transportation.

**Table 3.** Main physical-chemical features of the feedstock.

Parameter	Weight ratio (g/kg)	Mass ratio (%)	Concentration (mg/L)
Total solids, mean (SD)^a^	45 (3.15)	—^b^	—
Volatile solids, mean (SD)^a^	32 (3.28)	—	—
Total Kjeldahl nitrogen^c^	12.9	—	—
Phosphorous^c^	4	—	—
Chemical oxygen demand^c^	565	—	—
Chemical oxygen demand:nitrogen:phosphorous	—	100:2.2:0.7	—
Soluble chemical oxygen demand	—	—	25,814
N-NH_4_^+d^	—	—	325
P-PO_4_^3–e^	—	—	14
Volatile fatty acid^c^	—	—	3500
Volatile solids/total solids, mean (SD)^a^	—	72 (5)	—

a Based on 3 measurements.

b Not applicable.

c Measurements done for nitrogen, phosphor, and soluble chemical oxygen demand equivalents for total Kjeldahl nitrogen, phosphorous, chemical oxygen demand, and volatile fatty acid.

d N-NH_4_+: ammonium.

e P-PO_4_3-: phosphate.

### Statistical Analysis and Performance Indicators

#### Acidogenic Fermentation

**Figure 2.**
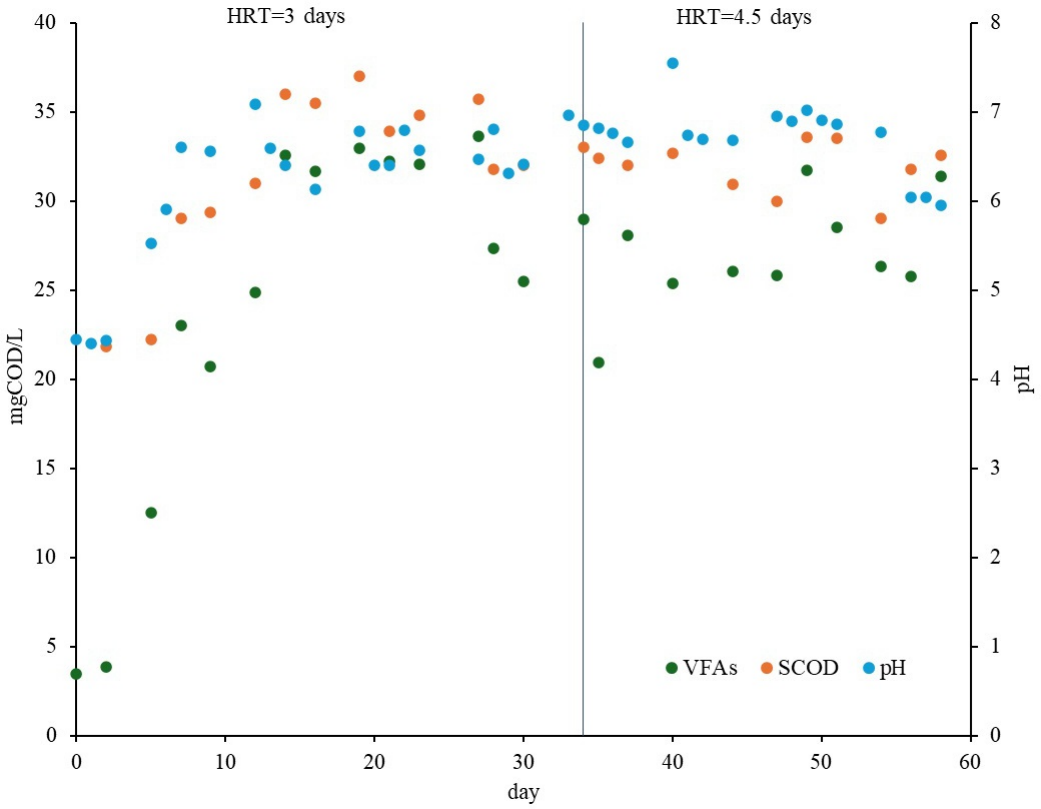
VFA, SCOD, and pH for mesophilic acidogenic fermentation. COD: chemical oxygen demand; HRT: hydraulic retention time; SCOD: soluble chemical oxygen demand; VFA: volatile fatty acid.

Table 4 presents the main physical and chemical characteristics of the effluent and solid cake from the acidogenic fermenters. According to Figure 2, the process reached a steady condition after 14 days, which was roughly 3 times the HRT (4.5 days). Both HRTs were similarly stable in terms of VFA concentration variation because of a negligible difference between SDs: 2.82 g-SCOD/L versus 2.45 g-SCOD/L. These values are less than 10% of the total VFA, and the VFA production continued for more than 3 weeks without any considerable issues. The lack of any change in this process is attributed to the initial high pH of 9‐10, which supported the process by keeping the pH variation in the optimal range of 6‐7.5 [32].

**Table 4.** Main physical-chemical features of the effluent and solid cake from mesophilic acidogenic fermentation.

Hydraulic retention time (days)	Total solids (g/kg), mean (SD)	Volatile solids (g/kg), mean (SD)	Volatile fatty acid (g–soluble chemical oxygen demand/L), mean (SD)	pH, mean (SD)
4.5^a^	43 (5.15)	23.6 (2.07)	30.77 (2.82)	6.56 (0.25)
3^b^	38 (4.55)	25.8 (1.5)	27.67 (2.45)	6.7 (0.45)

a 5 measurements for total solids and volatile solids; 9 measurements for volatile fatty acid; 13 measurements for pH.

b 4 measurements for total solids and volatile solids; 9 measurements for volatile fatty acid; 13 measurements for pH.

**Figure 3.**
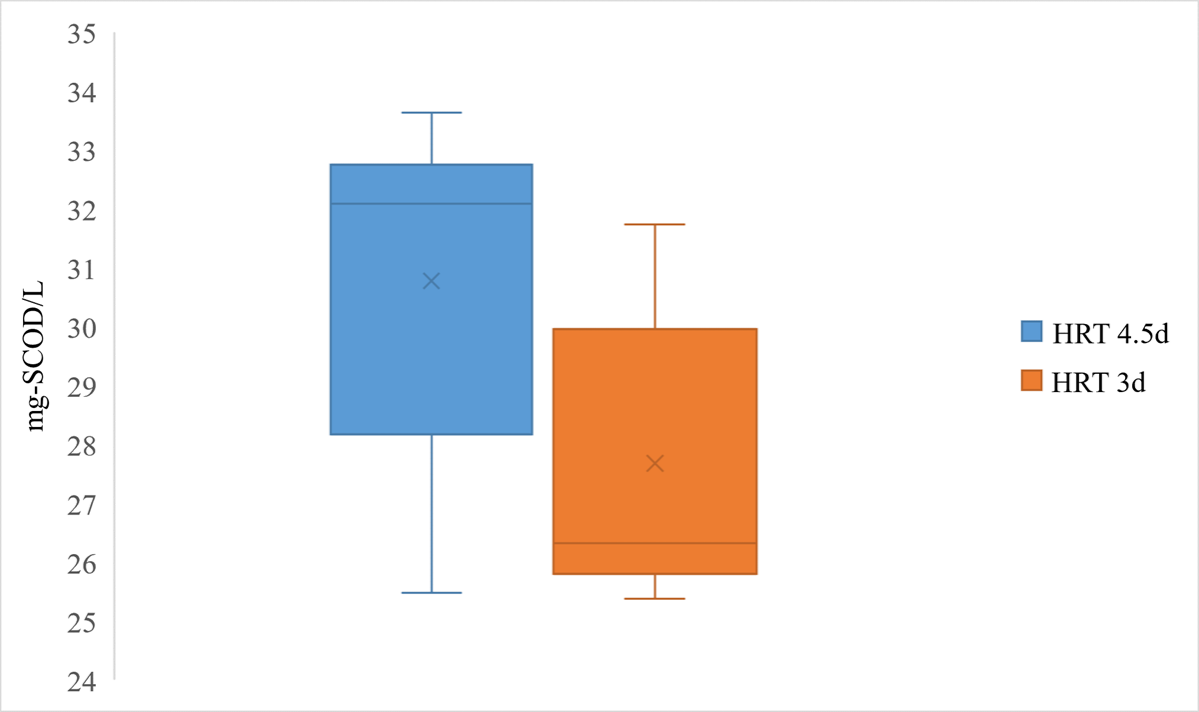
Box plot of volatile fatty acid concentrations for mesophilic acidogenic fermentation. HRT: hydraulic retention time; SCOD: soluble chemical oxygen demand.

Based on the *t* test results (*t*_8_=−2.68; *P*=.03; CI=95%), it was verified that the mean VFA concentration for an HRT of 4.5 days was significantly higher than the value for 3 days (30.77 vs 27.67 g-SCOD/L). A similar statistical analysis (*t*_8_=−0.99; *P*=.35; CI=95%) for the VFA/SCOD ratio rejected the significance of a higher mean value of 0.892 (SD 0.04) for an HRT of 4.5 days than 3 days, with a mean value of 0.87 (SD 0.058). The possible range of values for the VFA concentrations and VFA/SCOD, which cover 99% and 50% of the data for the 2 HRTs, are depicted by the box plots in Figures3  4, respectively.

**Figure 4.**
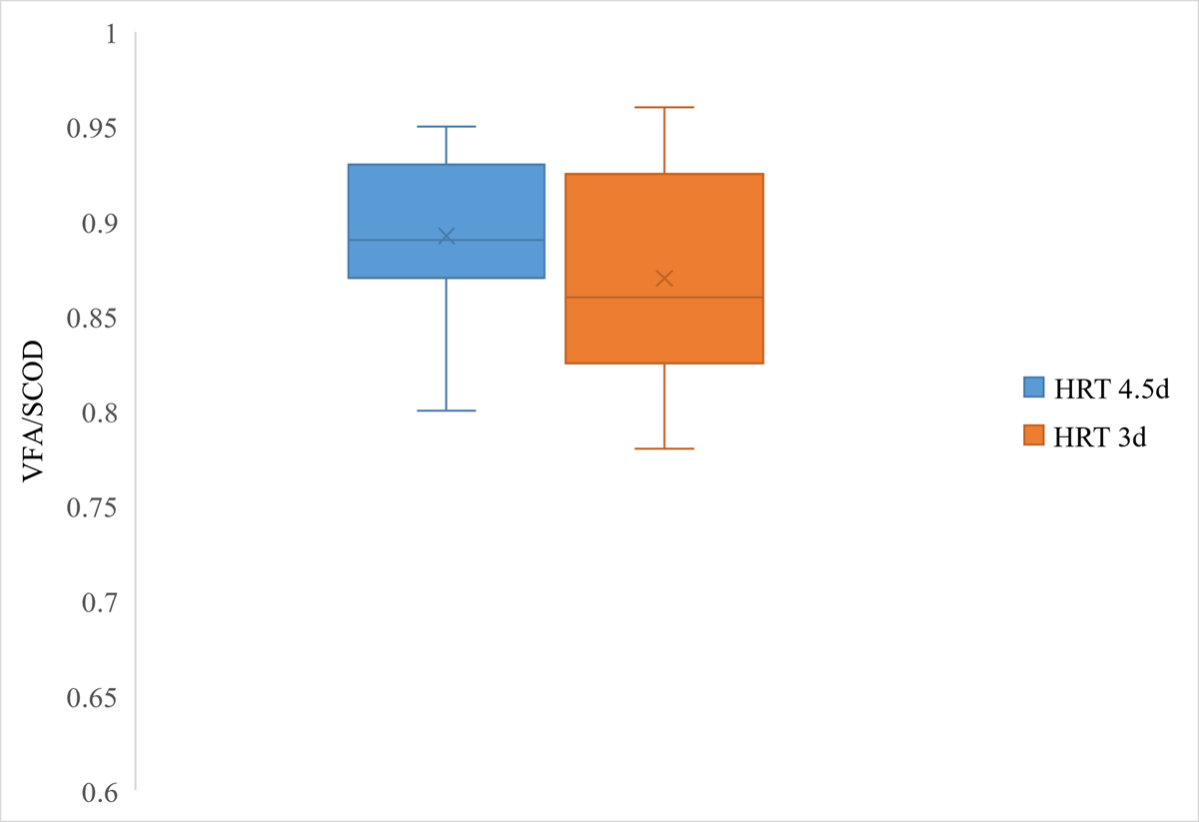
Box plot of VFA/SCOD ratios for mesophilic acidogenic fermentation. HRT: hydraulic retention time; SCOD: soluble chemical oxygen demand; VFA: volatile fatty acid.

Performance parameters for the 2 HRTs are given in Table 5. As can be seen, the HRT of 4.5 days gave higher COD solubilization and released more ammonia and phosphate than the HRT of 3 days. Moreover, the 0.57 VFA yield per gram of VS for the HRT of 4.5 days was significantly higher than 0.5 for the HRT of 3 days (*t*_8_=−2.94; *P*=.02; CI=95%).

In the biopolymer-synthesizing process, the aim was to generate a stable VFA weight ratio distribution with a high VFA/SCOD ratio for an efficient PHA synthesis during the whole process. Concisely, the VFA stream with a higher dominance of even numbers of carbon atom acids means a higher 3-hydroxybutyrate monomer synthesis compared to the 3-hydroxyvalerate, which is correlated with the net prevalence of odd numbers of carbon atom acids (propionic, valeric, and isovaleric acid) [33]. As can be inferred, the stability in the VFA spectrum means a predictable and reproducible PHA monomer production. Accordingly, the physical and mechanical features of synthesized biopolymers are stable [34,35].

**Figure 5.**
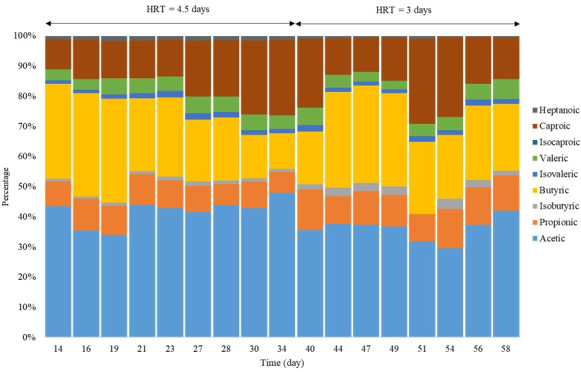
Volatile fatty acid weight ratio distribution for mesophilic acidogenic fermentation. HRT: hydraulic retention time.

Figure 5 reports the weight ratio distribution of the VFAs for the 2 HRTs. The main fractions were acetic acid (38%‐42%), butyric acid (24%), caproic acid (16%‐18.5%), propionic acid (9%‐11%), and valeric acid (5%). This VFA distribution, with a major part of butyric and acetic acid, is in line with those reported in similar studies [29,31]. In this respect, the VFA weight ratio distribution is determined by the type of feedstock and food waste rather than the operational conditions.

**Table 5.** Performance parameters of two different operational conditions used in mesophilic acidogenic fermentation.

Hydraulic retention time (days)	Solubilization (Δg–soluble chemical oxygen demand/g-VS^a^_0_), mean (SD)	Y_VFA^b^_ (Δg-VFA/g-VS_0_), mean (SD)	Ammonia release (%), mean (SD)	Phosphate release (%), mean (SD)
4.5^c^	0.28 (0.06)	0.57 (0.06)	35 (10.74)	13.7 (8.77)
3^d^	0.19 (0.05)	0.50 (0.06)	29 (0.11)	11 (0.06)

a VS: volatile solids.

b VFA: volatile fatty acid.

c 9 measurements for solubilization (*Y*_*VFA*_); 8 measurements for ammonia and phosphate release.

d 9 measurements for solubilization (*Y*_*VFA*_); 7 measurements for ammonia and phosphate release.

#### Anaerobic Digestion

**Figure 6.**
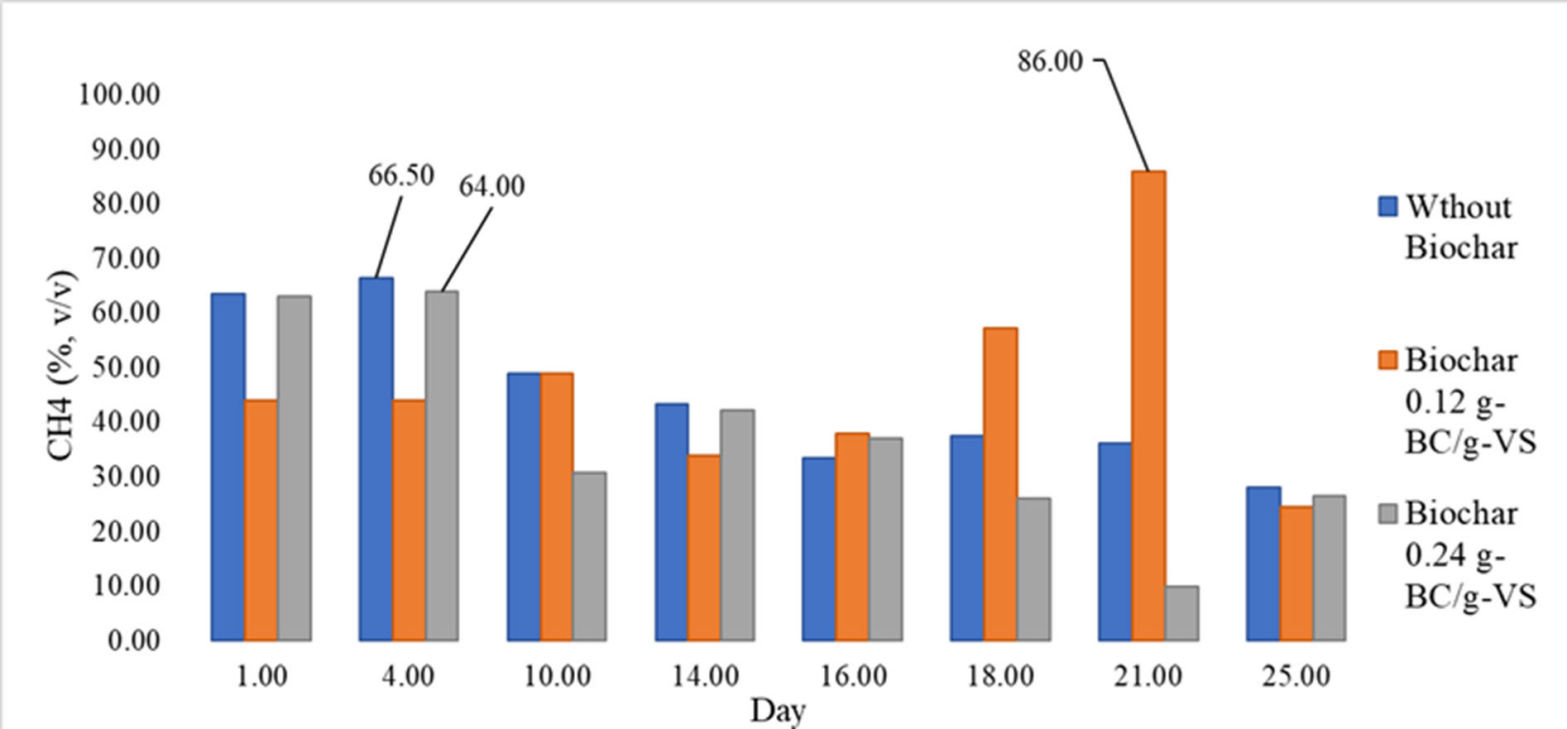
CH_4_ content in v/v for 3 different biochar dosages in anaerobic digestion. BC: biochar; CH_4_: methane; VS: volatile solids; v/v: volumetric basis.

Table 6 summarizes the performance parameters and the results from the kinetics study for anaerobic digestion. This study obtained a remarkably high value for the hydrolysis rate (ie, 0.58, 1/d) with no lag phase. Besides, a biogas yield of 0.61‐0.83 g-biogas/g-VS, SMP of 0.133‐0.204 CH_4_-Nm^3^/kg-VS, and an average composition of 45%‐58% methane (v-CH_4_/v-biogas) were obtained. According to Figure 6, adding biochar provided the desirable conditions for the growth of hydrogen using methanogenesis manifested through a higher maximum volumetric methane content (86% vs 66% volumetric basis [v/v]).

**Table 6.** The performance indicators for anaerobic digestion and results from the kinetics study for two models: (1) first-order rate and (2) modified Gompertz.

Experiments	Specific methane production (CH_4_^a^-Nm^3^/kg-VS^b^)	Specific gas production (CH_4_-Nm^3^/kg-VS)	K^c^ (1/d)	*R*_m_^d^ (CH_4_-mL/g-VS.d)	𝜆^e^ (days)	RMSE^f^ first-order (CH_4_-Nm^3^/kg-VS)	RMSE modified Gompertz (CH_4_-Nm^3^/kg-VS)	Max CH_4_ content (v/v^g^), %
Without biochar	0.204	0.540	0.57	76.12	0	10.4	6.82	68.5
Biochar (0.12 g-biochar/g-)	0.133	0.567	0.69	62.42	0	5.74	5.59	86
Biochar (0.24 g-biochar/g-)	0.177	0.500	0.58	65.17	0	9.64	3.39	76.5

a CH_4_: methane.

b VS: volatile solids.

c Hydrolysis rate.

d Maximum methane production rate.

e Lag phase.

f RMSE: root mean squared error.

g v/v: volumetric basis.

The mass flow model was adopted for 0.12 g-biochar/g-VS as the only feasible solution. Unlike other dosages, it could satisfy the assumptions for an FS/IN ratio of 0.3 at an HRT of 20 days, which was adequately long enough to let the methanogens reproduce themselves. Detailed information is available in the Excel sheet named “DIGESTER DESIGN” [27]. Besides, the high alkalinity of the biochar as reported in Table S1 in Multimedia Appendix 1 signifies a benefit of the biochar addition in limiting the concern about decreases in pH for a high OLR in full-scale implementation. Accordingly, almost 4-fold of the ordinary OLR was obtained, that is, 6.25 kg-VS/m^3^.d, by minimum water dilution, knowing that the biochar could maintain the stability of the process. Therefore, the digester volume will decline at the rate of 28 L/PE. Hence, the presented mass flow line model was implemented based on the results of 0.12 g-biochar/g-VS, the weighted average composition of biomethane as 35% v/v, and the SGP as 0.56 biogas-Nm^3^/kg-VS for an HRT of 20 days corresponding to an FS/IN ratio of 0.3.

Based on the root mean squared error reported in Table 6, both models were almost identical in describing biomethane production for a biochar dosage of 0.12 g-biochar/g-VS, and for simplicity, we used the first-order rate model in the feasibility study.

### Technical and Economic Assessment

Assuming an imaginary municipality of 70,000 PEs and the amount of TS production per capita as 0.3 kg/PE per day [36], the inlet to the scale-up line would be 21,000 kg-TS per day.

In the first scenario, the biowaste stream, after passing through the screw press and pretreatment unit, had a mass flow of 113,788 kg per day, TS of 4.1% kg/kg, and VS of 3.1% kg/kg. Then, the mixture was heated to 37 ℃ before and in the acidogenic fermenter, which was operated at an HRT of 4.5 days and OLR of 6.89 kg-VS/m^3^.d. This process was performed to convert biosolids into the VFAs and SCOD at concentration levels of 30.77 g-SCOD/L and 34 g-SCOD/L, respectively. At this step, the gaseous flow rate was assumed to be zero, as an HRT of 4.5 days is short for any adequate growth of methanogens in mesophilic conditions. The stream out of the acidogenic fermenter had a mass flow rate of 113,788 kg per day, with a VFA content of 3501 kg-SCOD per day, which could be used in the PHA-synthesizing step [37]. The outlet of this step was used in the separator to gain overflow and solid cake. Later, the solid cake was minimally diluted by water before being fed into a mesophilic anaerobic digester with a biochar addition of 0.12 g-biochar/g-VS. The anaerobic digester received a TS content of 18% kg/kg and a flow rate of 18,180 kg per day, corresponding to an HRT of 20 days and OLR of 6.25 kg-VS/m^3^.d. Overall, an SGP of 0.285 (Nm^3^-biogas/kg-VS) was obtained assuming zero gas production in acidogenic fermentation.

In the second scenario, the fresh feedstock, after being screw-pressed, had a mass flow rate of 4678 kg-TS per day and 28% kg/kg dry matter. Then, it was diluted with water and heated before being fed into the anaerobic digester. At this step, the mass flow rate of 85,012 kg per day with a TS of 6% kg/kg entered the digester with a volume of 2125 m^3^, leading to an HRT of 25 days and OLR of 1.7 kg-VS/m^3^.d. The SMP of 0.311 Nm^3^-biogas/kg-VS was obtained by destroying 80% of the VS.

**Figure 7.**
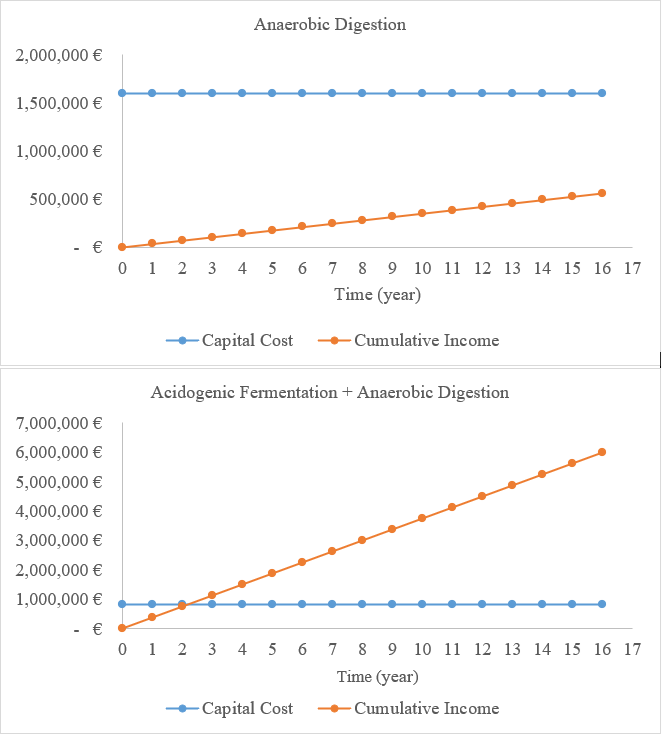
Capital cost and cumulative yearly income for the two proposed scenarios. A currency exchange rate of €1=US $1.05 is applicable.

In this study, working volumes of 512 m^3^ and 364 m^3^ were adopted for the acidogenic fermenter and anaerobic digester in the first scenario, respectively, and 2125 m^3^ for the anaerobic digester in the second scenario. As a result, the capital cost for the presented line was almost €809,000, roughly half of the quantity for the single-step anaerobic digestion (Figure 7). Unlike the single-step anaerobic digestion that converts all VS to biogas, this novel line shared the recovery of VS between higher added-value VFAs and biogas production, and expectedly generated 10-fold higher benefits (€375,085). Consequently, the payback period was reduced by more than 20 times in 2 years (Figure 7). This period was achieved using less surplus energy (2251 megajoules [MJ]/d) for the 2-step fermentation (vs 21,567 MJ/d for the single-step anaerobic digestion).

## Discussion

### Principal Results

We showed that multistep fermentation followed by anaerobic digestion is both economically and technically feasible. The findings indicated that producing VFAs and biogas in separate stages can significantly reduce the payback period for upcoming investments in biorefinery projects and result in the creation of a highly desired stream that is rich in VFAs. Additionally, the process stability could be maintained even at a high OLR by adding biochar and converting the VS’s easily biodegradable COD content into VFAs in the first phase. This would preserve energy and water, and reduce the digester’s volume.

### Comparison With Previous Studies

Because of the extra pretreatment unit in this research, the VFA yield of 0.57‐0.63 Δg-VFA/g-COD_IN_ was roughly double the value reported by Valentino et al [31] for the same OMSW.

Our results also indicate a substantial improvement in the process kinetics, which was manifested through a more than 8-fold rise in the hydrolysis rate (0.58 vs 0.07, 1/d) and a full decrease in the lag phase (0 vs 2.69 days) as opposed to the previous study by Karki et al [38]. This improvement is attributed to the destruction of the solids structure caused by bacterial enzymes and a hot alkaline solution. Additionally, a higher active biomass per feedstock was provided using a fine-tuned FS/IN ratio of 0.3 (VS basis), which was noticeably lower than the quantities (1 and 0.5) reported in similar studies [38,39].

The values for SMP and mean methane volumetric content presented in this study are lower than those reported by Valentino et al [29] (ie, 0.25 CH_4_-Nm^3^/kg-VS and 63%‐64% v/v, respectively). This difference is explained by the added fresh WS, which has a higher digestible content and better nutrient balance than the fermented solids. Similarly, the SMP in this study was lower than the 0.384 CH_4_-Nm^3^/kg-VS found in the study by Moreno et al [39]. This study investigated the anaerobic digestion of residual solids from two steps of bioethanol production and saccharification on OMSW. In this respect, bioethanol production can only convert part of the cellulose, starch, and some dissolved carbohydrates. Consequently, a great part of the biosolids’ volatile content, nearly 70%, is still available to be harvested in different biorefinery schemes compared with the one proposed in this method with 55%. Besides, the fermented OMSW would have a completely incompatible composition since it did not only come from different geographical locations (Spain and the United Kingdom) with different food habits but also underwent different biological pretreatment. Further, the multistep recovery line proposed in our study is more practicable technically. As the method studied by Moreno et al [39] requires sterilization conditions, imposing an additional operational cost and bioethanol concentration should be high enough to lower the cost of the subsequent distillation step.

Furthermore, our method for VFA production distinctively from biogas was preferable to the study by Papa et al [9], wherein the operational alteration on a single anaerobic digester was performed to obtain VFAs and biogas. These researchers asserted that the single-step recovery of biogas and VFAs was feasible by increasing the OLR while keeping the SMP of the reactor almost unaffected. The main recovery path for the VS was still biogas production in their study, which accounted for more than 90% of the VS conversion. Meanwhile, our study obtained 36% and 64% of the biogas and VFA conversion share, respectively. Further, whereas the destruction of VS of around 70% was achieved in both studies, their proposal limited the VFA distribution to propionic and butyric acid. The explanation is that some of the VFAs were converted into biogas in the same unit, which could negatively affect the PHA synthesis step later.

### Conclusion and Limitations

This paper demonstrated the technical and economic feasibility of a multistep recovery line for OMSW. The results of this study indicate that the production of VFAs and biogas in distinct steps can considerably shorten the payback period for future investments in biorefinery projects and produce a highly desirable VFA-rich stream. Further, adding biochar and converting easily degradable COD content in the VS into VFAs in the first step could maintain the process stability even with a high OLR in anaerobic digestion. As a result, it leads to energy and water preservation and a decrease in the digester volume. However, consideration should be paid to the full-scale implementation since the pilot studies cannot resemble the stability of the real process. For instance, operational alterations such as raising the OLR and the addition of biochar in the full-scale implementation might perturb the process pH or the synergetic balance between the bacterial communities and stop the process completely, which was never observed in our experimental study. Further, the superb profitability of the proposed line was highly variable because our cost analysis was too simplistic and did not elaborate on all the possible associated expenditures and incomes. Besides, since many of its components were from subject matter experts rather than the pilot studies’ budget, they were prone to site variations and uncertainties. Addressing the systematic uncertainty in the labor and material costs due to the changes in the supply chain issues, inflation, and site variations is beyond our scope. Moreover, caution should also be considered regarding the significance of the BMP results with the marginal difference since the number of samples was not large enough for statistical analysis. Nevertheless, the results presented in this study were prepared cautiously both technically and financially to encourage the revolution in the current state of organic waste valorization in Italy and any similar location.

In conclusion, a robust framework was proposed to assess the valorization of organic waste through experimental tests, statistical analysis, process kinetics, and mass and energy flow analysis. The findings support considerably higher profitability and, thus, a shorter payback period for the multistep fermentation than the current single anaerobic digestion. Additionally, our results encourage the circular economy perspective on converting OMSW into biogas and VFAs with the benefit of fewer residual solids due to reusing them in a pyrolysis line.

## Supplementary material

10.2196/50458 Multimedia Appendix 1 Supplementary table, equation, digester design, and code.

## Abbreviations

BMP: bio-methane potential
CH_4_: methane
CO_2_: carbon dioxide
COD: chemical oxygen demand
FS/IN: feedstock/inoculum
H_2_: hydrogen molecule
HRT: hydraulic retention time
kPa: kilopascal
MJ: megajoules
MWh: megawatt-hour
N-NH_4_^+^: ammonium
N_2_: nitrogen molecule
NaOH: sodium hydroxide
O_2_: oxygen molecule
OLR: organic loading rate
OMSW: organic municipal solid waste
P: phosphorous
P-PO_4_^3-^: phosphate
PE: population equivalent
PHA: polyhydroxyalkanoate
rpm: rounds per minute
SCOD: soluble chemical oxygen demand
SGP: specific biogas production
SMP: specific methane production
TS: total solids
v/v: volumetric basis
v/v %: maximum volumetric methane content
VFA: volatile fatty acid
VS: volatile solids
WS: waste sludge
WWTP: wastewater treatment plant
